# SB Brasil Project: brief history and perspectives for the 2030 edition

**DOI:** 10.1590/1980-549720260015.supl.1

**Published:** 2026-07-31

**Authors:** Paulo Capel Narvai

**Affiliations:** I Universidade de São Paulo, School of Public Health, Department of Policy, Management, and Health – São Paulo (SP), Brazil.

**Keywords:** DMF index, Oral health, Health survey, Calibration, Field epidemiology

## Abstract

**Objective:**

To analyze the evolution of population-based epidemiological surveys in oral health (saúde bucal) in Brazil, and implications for the 2030 edition of the SB Brasil project, of the Ministry of Health.

**Methods:**

This is an essay problematizing methodological and management aspects, and their implications for fieldwork, of population epidemiological surveys in oral health carried out in Brazil in different years, from 1986 to 2023.

**Results:**

Methodological and management decisions of the editions of the SB Brasil project, including 2023, are analyzed, focusing on the inclusion and exclusion of variables, such as dental fluorosis and the PUFA index, which registers the presence of seriously damaged teeth and which have visibly compromised pulp (P), ulcers caused by fragments of loose teeth (U), fistula (F) and abscess (A). The list of variables and the sampling plans of the nationwide oral health surveys carried out in 1986, 1996, 2003, 2010, and 2023 are problematized. The consequences of considering only clinical interest as a criterion for inclusion and exclusion of variables from epidemiological survey protocols are discussed, and the difficulties for the calibration of examiners are addressed, even when standardized methods are used.

**Conclusion:**

The next editions of the SB Brasil institutional project, of the Ministry of Health, will start from a relevant accumulation of knowledge and practices resulting from the Brazilian experience with oral health epidemiology, within the scope of SUS, developed in the second half of the 20th century and the first decades of the 21st century.

## INTRODUCTION

The Oral Health Brazil project (SB Brasil) comprises a series of population-based epidemiological surveys conducted by the Ministry of Health, which began in 2000. It is an institutional project, with editions scheduled to be carried out every ten years. Following the completion of three editions in 2003, 2010, and 2023, only the 2010 edition was executed in accordance with the established schedule. The 2003 edition was originally scheduled for completion in 2000, while the 2023 edition should have been completed in 2020.

Joining the legacy of these three editions are the pioneering “Epidemiological Survey on Oral Health: Brazil, Urban Areas, 1986”^1^ and the “Epidemiological Survey on Oral Health 1996: Dental Caries”^2^. Together, they constitute a comprehensive archive comprising five distinct databases, which represents a formidable foundation of data regarding oral health conditions. Unprecedented in scope on a global scale, this archive has given rise to hundreds of scientific articles published both within Brazil and internationally. Plans are currently being considered to conduct a further edition of the SB Brasil project in 2030.

This article aims to contribute to an understanding of the history behind the creation of the SB Brasil project, as well as its institutional characteristics. It begins by examining historical aspects of the Brazilian experience regarding oral health epidemiology, followed by an analysis of the technical-scientific and operational facets of the project’s first three editions. The article concludes with a set of considerations and recommendations regarding which elements should be retained and which should be modified in future editions, with a specific focus on the feasibility and viability of SB Brasil as an institutional project.

### The who and the epidemiology of oral health

Shortly after the creation of the World Health Organization (WHO) in 1948, efforts began to establish standardized methods for obtaining epidemiological data on oral health, particularly regarding dental caries. The primary concern lay with the measurement and classification of prevalence, and as by the mid-20th century, dozens of measurement instruments had been proposed to assess the status of this disease within populations^3^. The indices and coefficients proposed in the literature focused at times on the type of lesion (site, enamel, dentin, pulp) and at other times on the unit of measurement (lesion, surface, tooth). The DMF or dmf index, referring to decayed-missing-filled, proposed in 1937 by Klein & Palmer^4^, had not yet attained the popularity that would render it a “classic” in the decades that followed.

In the 1960s, the WHO consolidated global expertise in caries epidemiology and —adopting the DMF index as a foundation, and the tooth as the unit of measurement (DMFT) — published the first manuals establishing standards for assessing dental caries in populations^5^. Dating from this period are two official WHO publications that constitute a landmark reference for population-based epidemiological research in oral health. In 1962, the *Standardization of reporting of dental diseases and conditions* (Technical Report Series, No. 242) was published, containing “definitions and criteria for measuring the prevalence of dental caries, gingivitis, periodontal disease, dentofacial anomalies, dental eruption, and prosthetic needs”^6^. This initiative was hailed by the journal Nature, which, in an editorial note, emphasized that these were “methods recommended primarily for large-scale studies of differences between populations”^7^. In 1969, the WHO published the first edition of the International Classification of Diseases: Application to Dentistry and Stomatology^8^, which marked the first inclusion of oral health conditions within the International Classification of Diseases the ICD-8.

**Figure 1:**
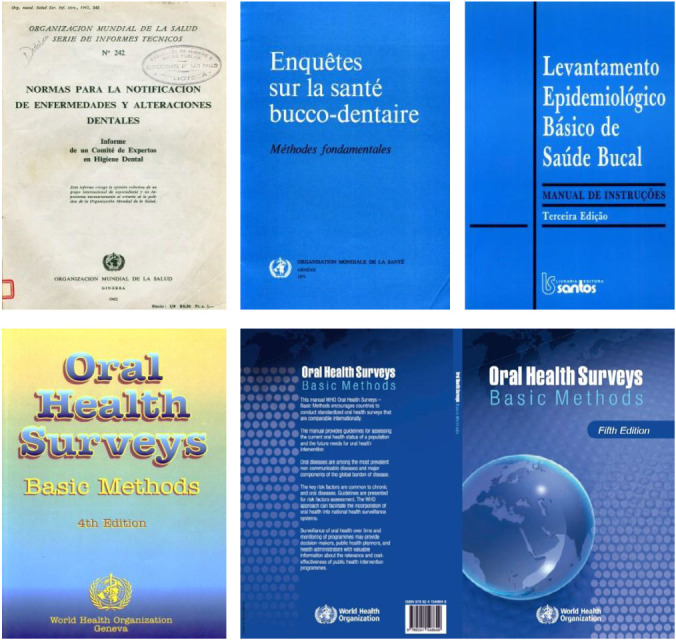
Covers of different editions, in various languages, of the WHO instruction manual for basic epidemiological surveys in oral health.

Beginning in the 1970s, the WHO periodically updated this publication, which it titled “Basic oral health survey: a manual of instructions” (Figure 1), providing guidelines for planning such research and standardizing a methodology that would enable comparisons across time and space. In the specific section regarding the dental crown, it proposed what would later become known as the “WHO combined method”, in which the condition of the crown and the type of treatment need associated with that specific condition are evaluated simultaneously. The 5th edition, the most recent version of this manual, was released in 2013^9^.

### The brazilian experience with oral health epidemiology

The first records of epidemiological interest regarding oral health in Brazil emerged in the mid-20th century through studies in the field of social sciences. These consisted of reports offering general descriptions of the dental conditions of the native population. A professor of dental prosthetics at New York University took particular note of the low incidence of dental caries in certain regions of the Brazilian Northeast^10^.

However, it was only with the first school dentistry programs, implemented by the Special Public Health Service in the Rio Doce Valley (spanning Minas Gerais and Espírito Santo) and other strategic locations across Brazil’s various regions, that the first “caries surveys” appeared. These surveys were conducted annually within school settings, covering the population aged 6 to 14 and utilizing the DMF index^11^.

Beginning in the 1990s, in the state of São Paulo, specifically in municipalities with larger populations and greater socioeconomic heterogeneity, such as the state capital and the coastal cities of Santos (1995) and São Vicente (1996), more comprehensive oral health surveys were carried out, extending their scope to include students in private schools^12^. In 1998, the state of São Paulo conducted a statewide survey^13^. Similar initiatives followed in several other states (such as Minas Gerais and Paraná) and state capitals, including Florianópolis, with the EpiFloripa project^14^, and Goiânia^15^. In these surveys, specific sampling plans were devised, or the sample size was expanded beyond that of a standard edition of the SB Brasil survey.

### The who-fdi oral health goals for 2000

To assess the status of dental caries in Brazil 10 years after the 1986 “survey,” the Ministry of Health conducted a survey in 1996 retaining the “WHO combined method” but employing a “simplified” sampling plan suggested by the WHO for resource-constrained countries. Brazilian experts deemed this simplification inappropriate for the country. Data collection encountered difficulties in several municipalities, including state capitals^2^. The Ministry of Health sought to prepare itself to conduct, in the year 2000, an epidemiological survey to evaluate the attainment of the Oral Health Goals established for that year by the WHO^16^.

The “WHO-FDI Goals/2000” had been proposed to the WHO in 1981 by the World Dental Federation (FDI)^17^. Although the WHO accepted the FDI’s proposal, criticisms soon emerged, arguing that it made no sense for the WHO to propose global goals^18^. Nevertheless, the WHO-FDI Goals 2000 were used in public health planning and evaluation^19^, leading even to the consideration of new goals for the year 2010^20^. However, these were never formally established.

### Repibuco and creation of the sb Brasil project

In July 1996, 58 dental professionals with an interest in oral health epidemiology, gathered in Curitiba during the 12th National Meeting of Administrators and Technicians of Public Dental Services (Enatespo), noted the insufficient volume of Brazilian research in this field. Even regarding dental caries, basic information concerning the status of the disease in major municipalities across the country’s various regions was unavailable. For other oral health issues, population-based data was either nonexistent or of poor quality. They therefore decided to propose to the Ministry of Health the creation of a Brazilian Network for Oral Health Epidemiology (Repibuco). Among the objectives were: to create and maintain a nationwide database on oral health epidemiology in Brazil; and to help facilitate the execution, in the year 2000, of a nationwide, population-based epidemiological survey in this field. However, the Brazilian government at the time rejected the proposal and opted for a different strategy^21^.

Following the unsuccessful experience of 1996^2^ and as the year 2000 approached, epidemiologists affiliated with Repibuco, along with various dental organizations and leaders, inquired of the Ministry of Health as to how Brazil intended to address the WHO-FDI Goals for the year 2000. These inquiries prompted the Ministry of Health to establish the SB Brasil project and schedule its first edition for the year 2000, to be conducted within the framework of the Unified Health System (SUS).

The SB Brasil 2000 survey faced a particularly difficult implementation process and was not completed until 2003^22^. The 2010 and 2020 editions followed thereafter.

### The 2023 edition of sb Brasil

No population-based epidemiological survey is free from difficulties of various kinds^23^; these difficulties, in each study, contribute in some way to the refinement of methods and techniques, thereby advancing knowledge in this field. SB Brasil — as an institutional project linked to SUS — serves as a prime example. The 2003 edition led to improvements in sampling designs^22^. From the 2010 edition, ethical issues emerged stemming from the nature of the examiner calibration training — a technique that requires the repetition of examinations on the same individual^24^. Prior to these editions, neither sampling design nor the ethical aspects of calibration had been given sufficient emphasis within the SB Brasil project. In the 2023 edition, these aspects received due attention, and the outcome was positive.

The 2020–2023 edition was marked by an unfavorable federal institutional context and the COVID-19 pandemic. Furthermore, it saw significant changes compared to the 2003 and 2010 editions regarding research management, as well as methodological alterations that impacted fieldwork. These changes gave rise to difficulties of various kinds — issues that warrant analysis with a view toward future editions of SB Brasil. Although the emphasis of this article lies on the most recent edition (2023) of the SB Brasil project, due consideration must be given to the procedural aspect that characterizes SB Brasil and constitutes the subject of this analysis.

### Research Management

The editions completed in 2003 and 2010 were planned and executed by a technical-scientific coordination body established by the Ministry of Health. Its activities were managed by the General Coordination of Oral Health with support from partner entities, operating under agreements with the Ministry, specifically for the management of financial resources allocated to the research. In 2000, the partner was the Brazilian Dental Association (ABO), and in 2010, the University of São Paulo (USP). Nevertheless, there was no doubt that SB Brasil was a research initiative conducted by the Ministry of Health within the scope of SUS and, therefore, constituted a shared responsibility among all federative entities^25^.

However, the 2023 edition altered this pattern, with the Ministry of Health entrusting the Federal University of Minas Gerais (UFMG) with not only the financial management of research funds but also their technical-scientific coordination. This decision had implications for the public image of the SB Brasil, which was often viewed as “a UFMG research project”. It was not enough that faculty members affiliated with UFMG — as well as those from other universities participating in the SB Brasil — consistently denied that this constituted an “outsourcing of research”, for that is precisely how the SB Brasil 2023 was perceived by many SUS professionals.

### Methodological characteristics

Decisions involving changes to the standardization adopted by the SB Brasil project, based on the “WHO combined method”, are not simple, as they entail methodological considerations of various orders. Any exclusion — such as that of dental fluorosis, or inclusion, such as that of PUFA, should stem from exhaustive analyses of these implications, involving various specialists with epidemiological expertise in the decision-making process. The way this decision-making process unfolded, culminating in the outcome we now know, is a subject that warrants further thought.

In the case of the exclusion of dental fluorosis, the main arguments made public are tenuous (low prevalence; concentration of cases in endemic areas; lack of status as a relevant public health issue; extreme difficulty in calibration), and they do not adequately justify the decision for the following reasons:

a) Epidemiological surveys are conducted to capture the current situation, but they also serve as a basis for comparisons over time. Thus, considering only the current prevalence of an anomaly that has garnered global interest does not justify its exclusion.
b) The concentration of cases in endemic areas is a characteristic of “chronic endemic” dental fluorosis; however, this is not the only type of dental fluorosis that triggers alerts within epidemiological surveillance systems across various countries. In areas where exposure does not stem from a single source of fluoride, but rather from multiple sources, including toothpaste, “iatrogenic endemic” dental fluorosis^26^ is the manifestation of the anomaly that holds the greatest significance in population-wide surveys of national scope, such as the SB Brasil.
c) Public health issues are dynamic; over time, they may gain or lose relevance depending on various factors beyond the control of epidemiologists. For this reason, measurement (rather than non-measurement) should be the default option in such situations.
d) The calibration of examiners to assess the presence of dental fluorosis (using Dean’s Index) — while admittedly challenging — can be effectively addressed through well-planned and well-executed training programs. In the 2010 edition of the SB Brasil, we proposed “in lux” calibration as a means to enhance training, aiming to mitigate and overcome the subjectivity inherent to the index^24^. The results, however, were unsatisfactory. Nevertheless, this outcome was not attributable solely to the inherent subjectivity of the index or to calibration difficulties; rather, as documented in the scientific literature, it stemmed from poorly planned and executed calibration training sessions, and even from data falsification during fieldwork, which necessitated a complete re-collection of the data^25^.

The inclusion of the PUFA index represents the opposite of what occurred with dental fluorosis: its inclusion should not have taken place. Since the mid-20th century — when the WHO convened oral health epidemiologists in an effort to standardize the methods to be employed in epidemiological surveys — a consensus has been established regarding the importance of population-based studies considering data reliability, derived from both precision and validity. In this context, the sensitivity, specificity, and predictive value required of the instruments (indices) to be utilized are decisive^27^. Therefore, even though there is significant interest in estimating the prevalence of certain clinical conditions, caution must be exercised when including them in broad-scope population-based surveys, ensuring that their inclusion is adequately justified^28^. Prevalence data related to conditions that are often transient, e.g., mucosal lesions, and due to their inherent characteristics, the data are considered “problematic”^29^ and “difficult to compare with findings from previous studies”^30^. Given their limitations in prevalence studies — specifically, the pronounced intra- and inter-examiner disagreements that diminish reliability and render examiner calibration more complex and highly demanding^28^ — the epidemiological instruments developed to assess such conditions should instead be used in longitudinal epidemiological studies^30,31^. Furthermore, the inclusion of the PUFA index constituted an unnecessary duplication of data, given that the “combined method” is sufficient for the epidemiological assessment of tooth loss and “untreated caries”. The authors who proposed the PUFA index^32^ reiterate arguments regarding “limitations of the DMFT index”, arguments that had already been rejected by oral health epidemiologists with field experience, as early as the mid-20th century^33^.

### Aspects of fieldwork

Beyond the inherent difficulties of the territories where data are collected, fieldwork is strongly affected by two components that require special attention: calibration training and the set of variables contained in the form, or “examination sheet”.

The emergence of the COVID-19 pandemic imposed severe restrictions on calibration work, which was subsequently carried out using Digital Information and Communication Technologies (ICT)^34^. The use of ICT constituted a significant advancement in this edition of the SB Brasil, enhancing training and addressing ethical aspects of this fieldwork preparation phase, notably the discomfort experienced by volunteers regarding the need for repeated examinations^24^.

However, the primary issue affecting the fieldwork was the sheer number of variables, namely, 413. This excessive number entails increased costs. Underlying this problem is the inclusion of variables suited for longitudinal studies but not for prevalence studies (such as PUFA), as well as the duplication of collected data. Here are just two examples of such duplication: 1) “dental trauma” could be assessed as a single category (or condition) within the “dental crown” variable; and 2) “dental occlusion” could be assessed using a single variable with three categories or, where appropriate, by utilizing solely the Dental Aesthetic Index (DAI). Although the inclusion of these variables in the SB Brasil 2023 edition was based on the 5th edition of the WHO Manual, the consequences of this decision should be prioritized during the planning of the 2030 edition. The guiding principle for the 2030 edition should be a clear conceptualization of what a survey like the SB Brasil should be, specifically by addressing the central question inherent to this type of epidemiological research, a question that has been raised since the mid-20th century^3^: “What is the purpose of this survey?”.

### Data Availability Statement

The entire dataset supporting the results of this study, as well as access to the reports from the three editions of the SB Brasil Project and other mentioned institutional reports, may be obtained from the Ministry of Health, General Coordination of Oral Health.

### What to expect from sb Brasil 2030

**Figure 2:**
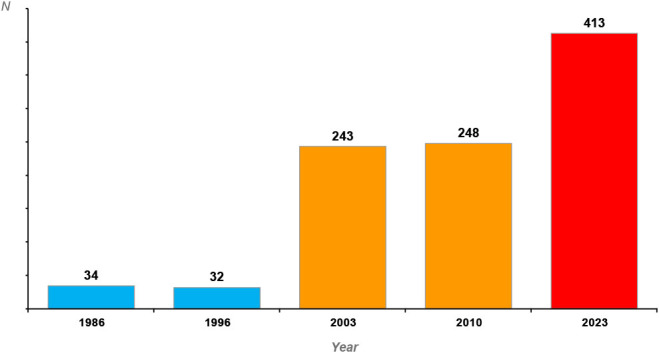
Number of variables in databases produced by epidemiological oral health population surveys in Brazil, in different years.

The feasibility of a 2030 edition of the SB Brasil will be evaluated over the next three years; however, even though our expertise points to a positive scenario, its practicability — facilitated by the accumulation and consolidation of Brazilian experience in this field — must be analyzed with urgency. One of the decisive aspects regarding practicability is reducing the number of variables comprising the “examination sheet” by using consolidated indices. This consideration stems from the observation that, with each successive edition, the number of variables and their respective categories has increased, as illustrated in Figure 2.

Brazil currently finds itself in a situation vastly different from that noted 30 years ago at the Enatespo conference in Curitiba. Over this period, the country has amassed a body of knowledge of inestimable value regarding the oral health conditions of its population. It is understandable, therefore, that professionals in this field seek to further refine their investigative processes, develop new methods, and enhance their techniques. It is indeed tempting to wish to include the maximum possible number of observable conditions within research protocols; however, parsimony, caution, and prudence are essential to avoid compromising the practicability of the SB Brasil and thereby jeopardizing its continuity as a valuable institutional project of the SUS.

In this regard, it is crucial to carefully consider costs, timelines, examination sites and techniques, the characteristics of the specific territories involved, and their implications for sampling plans, among other aspects related to the design of each SB Brasil edition. For this reason, it is critical to appropriately address the imperative of integrating clinical practice with epidemiology. Clinical practice must not be compromised, for, as the adage goes: “without clinical practice, there is no epidemiology”. Conversely, however, clinical practice must not disregard the non-clinical requirements inherent to population-based epidemiological research. These requirements are neither few nor simple.

**Figure 3:**
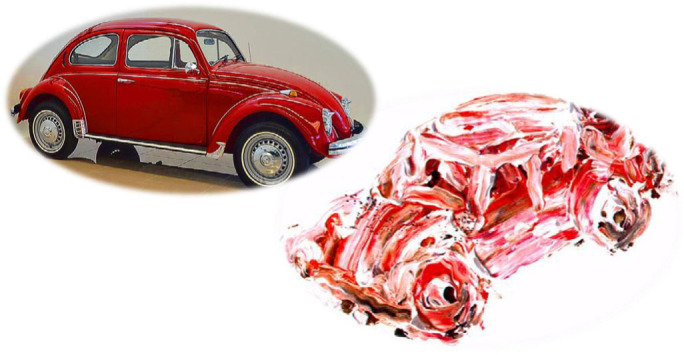
Two “red beetles”: one captured by a digital camera, and the other painted in the impressionist style by visual artist Sergio Romagnolo.

Frequently, there is an expectation that population-based surveys should produce an image of a population’s health-disease reality, an image possessing the highest possible degree of clarity and precision, mirroring in every minute detail the quality of a digital photograph. This would then constitute good epidemiology, the “faithful expression of reality”. More often than not, however, the most one can achieve is to produce an impressionistic picture. The encouraging aspect of population-based epidemiological research is that, even with the limitations of an impressionistic canvas, the image it produces can be sufficient to enable comparisons across time and space, and to guide decision-making. For this reason, epidemiological surveys also serve as strategic tools enabling administrators to fine-tune healthcare delivery models^12,23^. Figure 3, featuring both a photograph and a painting, illustrates this perspective. To a trained eye, a digital photograph is not essential; one can extract from the impressionistic painting, notwithstanding its “distortions” of reality, precisely what matters for assessing situations and guiding optimal decision-making.

## FINAL CONSIDERATIONS

The primary conclusion of the present analysis is that the composition of the technical-scientific coordination for each edition of the SB Brasil project, as well as the management of its activities, falls under the purview of the Ministry of Health. This includes defining the specific purpose of this type of epidemiological survey, thereby distinguishing it from longitudinal epidemiological studies in oral health. All professionals involved in the research, at every level, must share the understanding that the data to be collected are intended for epidemiological analysis rather than for clinical-therapeutic purposes.

Brazil’s extensive and consistent experience in this field warrants recognition and appreciation. In this domain, Brazil ranks among the world’s leading nations; this status necessitates timely and pertinent institutional actions to ensure that the expertise acquired over the past decades is not lost.

Aspects regarding the viability and feasibility of the upcoming edition were analyzed in this article, with particular emphasis placed on the need to reduce the number of conditions to be observed — thereby decreasing the number of variables compared to the 2023 edition — and on ensuring that only well-established epidemiological instruments are included. Surveys of the magnitude of SB Brasil, where data collection takes place across diverse geographic territories and under varying operational conditions, are not suitable venues for experimentation, as such trials can negatively impact sampling designs, costs, timelines, field sites, and examination techniques. The analysis presented herein underscores that decisions involving changes to the standardization framework established across the various editions of the SB Brasil project, which is grounded in the “WHO combined method”, are by no means simple matters. Such decisions carry methodological consequences of various orders, particularly those pertaining to the sensitivity, specificity, and predictive value required of the epidemiological instruments employed, given the critical implications of these factors for the overall reliability and validity of the resulting data.
