# Supplementary material for: SB Brasil Project: brief history and perspectives for the 2030 edition
Source: Rev Bras Epidemiol. 2026 Jul 31;29(Suppl 1):e260015supl1. doi: 10.1590/1980-549720260015.supl.1 (PMC13427313; doi:10.1590/1980-549720260015.supl.1)
Supplement: Translation

# Translation: Projeto SB Brasil: breve histórico e perspectivas para a edição 2030

**DOI:** 10.1590/1980-549720260015.supl.1.2

**Authors:** Paulo Capel Narvai

**Affiliations:** I Universidade de São Paulo, Faculdade de Saúde Pública, Departamento de Política, Gestão e Saúde – São Paulo (SP), Brasil.

**Keywords:** Índice CPOD, Saúde bucal, Inquérito epidemiológico, Calibração, Epidemiologia de campo

## Abstract

**Objetivo:**

Analisar a evolução dos inquéritos epidemiológicos de base populacional na área de saúde bucal no Brasil e as implicações para a edição 2030 do projeto Saúde Bucal (SB) Brasil, do Ministério da Saúde.

**Métodos:**

Trata-se de um ensaio problematizando aspectos metodológicos e de gestão, e suas implicações para o trabalho de campo, de inquéritos epidemiológicos populacionais na área de saúde bucal realizados no Brasil em diferentes anos, no período de 1986 a 2023.

**Resultados:**

São analisadas decisões de ordem metodológica e de gestão das edições do projeto SB Brasil, incluindo a de 2023, com foco na inclusão e exclusão de variáveis, como fluorose dentária e o índice PUFA, que registra a presença de dentes gravemente cariados em que há visível comprometimento pulpar (P), ulceração causada por fragmentos dentários deslocados (U), fístula (F) e abscesso (A). São problematizados o elenco de variáveis e os planos amostrais dos inquéritos de saúde bucal, de abrangência nacional, realizados em 1986, 1996, 2003, 2010 e 2023. Discutem-se as consequências de considerar apenas o interesse clínico como critério para inclusão e exclusão de variáveis de protocolos de inquéritos epidemiológicos, e são abordadas as dificuldades para a calibração de examinadores, ainda quando métodos padronizados são utilizados.

**Conclusão:**

As próximas edições do projeto institucional SB Brasil, do Ministério da Saúde, partirão de um acúmulo relevante de conhecimentos e práticas decorrentes da experiência brasileira com epidemiologia da saúde bucal, no âmbito do Sistema Único de Saúde (SUS), desenvolvidos na segunda metade do século XX e primeiras décadas do século XXI.

## INTRODUÇÃO

O projeto Saúde Bucal Brasil (SB Brasil) corresponde a um conjunto de inquéritos epidemiológicos de base populacional, realizados pelo Ministério da Saúde (MS), que se iniciou em 2000. Trata-se de um projeto institucional, cujas edições são previstas para serem executadas a cada dez anos. Após a realização de 3 edições concluídas em 2003, 2010 e 2023, apenas a de 2010 foi executada em conformidade com o cronograma. A edição de 2003 tinha a sua conclusão prevista para o ano 2000 e a de 2023 deveria ter sido concluída em 2020.

Ao legado dessas três edições, juntam-se o pioneiro “Levantamento epidemiológico em saúde bucal: Brasil, zona urbana, 1986”^1^ e o “Levantamento epidemiológico em saúde bucal 1996: cárie dental”^2^ para constituir um acervo, cujos cinco bancos de dados representam uma formidável base de dados sobre condições de saúde bucal que, sem precedentes em escala mundial, deu origem a algumas centenas de artigos científicos publicados no Brasil e em outros países. Cogita-se, para 2030, a realização de mais uma edição do SB Brasil.

Este artigo busca contribuir para a compreensão da história da criação e das características institucionais do projeto SB Brasil. Parte-se de aspectos históricos da experiência brasileira relacionada com a epidemiologia da saúde bucal e analisam-se aspectos técnico-científicos e operacionais das suas primeiras três edições. Conclui-se com considerações e recomendações sobre o que manter e o que mudar nas edições futuras, relacionadas com a factibilidade e a viabilidade do SB Brasil como projeto institucional.

### A oms e a epidemiologia da saúde bucal

logo após a criação da Organização Mundial da Saúde (OMS), em 1948, começaram os esforços para definir uma padronização de métodos para obtenção de dados epidemiológicos sobre saúde bucal, sobretudo a cárie. A principal preocupação era com a mensuração e a classificação da prevalência, pois em meados do século XX havia dezenas de instrumentos de medida propostos para avaliar a situação dessa doença em populações^3^. Os índices e coeficientes propostos na literatura ora focavam no tipo de lesão (local, esmalte, dentina, polpa), ora na unidade de medida (lesão, superfície, dente). O índice CPO, proposto em 1937 por Klein & Palmer^4^, ainda não tinha a popularidade que o tornaria um “clássico” nas décadas seguintes.

Nos anos 1960, a OMS reuniu a experiência mundial com epidemiologia da cárie e, adotando como base o índice CPO, e o dente como unidade de medida (CPOD), publicou os primeiros manuais estabelecendo padrões para aferir cárie em populações^5^. São desse período duas publicações oficiais da OMS que constituem um marco referencial para as pesquisas epidemiológicas de base populacional em saúde bucal. Em 1962, foi publicada a “Padronização da notificação de doenças e condições dentárias” (*Standardization of reporting of dental diseases and conditions – Technical Report, Series, 242*), contendo “definições e critérios para medir a prevalência de cárie dentária, gengivite, doença periodontal, anomalias dentofaciais, erupção dentária e necessidades protéticas”^6^. A iniciativa foi saudada pela revista *Nature*, que, em nota editorial, enfatizou tratar-se de “métodos recomendados principalmente para estudos em larga escala de diferenças entre populações”^7^. Em 1969, a OMS publicou a primeira edição de *International classification of diseases: application to dentistry and stomatology* (Classificação Internacional de Doenças: aplicação à odontologia e à estomatologia)^8^, que assinalou a primeira presença de condições de saúde bucal na Classificação Internacional de Doenças, a CID-8.

A partir dos anos 1970, periodicamente, a OMS atualizou sua publicação a qual denominou de “Levantamento epidemiológico básico de saúde bucal: manual de instruções” (Figura 1), contendo orientações sobre o planejamento desse tipo de pesquisa e padronizando um método que possibilitasse comparações no tempo e no espaço. Na parte específica sobre a coroa dentária, propôs o que viria a ser conhecido como “método combinado da OMS”, em que simultaneamente se avalia a condição da coroa e o tipo de necessidade associado à respectiva condição. A 5^a^ edição, versão mais recente desse manual, veio a público em 2013^9^.

**Figura 1:** Capas de diferentes edições, em diversas línguas, do manual de instruções da OMS para levantamentos epidemiológicos básicos em saúde bucal.

### A experiência brasileira com epidemiologia da saúde bucal

Os primeiros registros de interesse epidemiológico em saúde bucal no Brasil foram feitos, em meados do século XX, por estudos no campo das ciências sociais. Tratavam-se de relatos descrevendo, genericamente, condições dentárias da população nativa. A um professor de prótese dentária, da Universidade de Nova York, chamou a atenção a baixa incidência da cárie em certas regiões do Nordeste brasileiro^10^.

Entretanto, foi apenas com os primeiros programas de odontologia escolar executados pelo Serviço Especial de Saúde Pública (Sesp), no Vale do Rio Doce (Minas Gerais e Espírito Santo) e em outras localidades estratégicas nas diferentes regiões brasileiras, que vieram os primeiros “levantamentos de cárie”, realizados anualmente em unidades escolares, abrangendo a população de 6 a 14 anos de idade e utilizando os índices ceod e CPOD^11^.

A partir dos anos 1990, no estado de São Paulo, em alguns municípios de maior porte demográfico e maior heterogeneidade socioeconômica, como a capital e as cidades litorâneas de Santos (1995) e São Vicente (1996), foram realizados inquéritos de saúde bucal mais abrangentes, incluindo escolares da rede particular^12^. Em 1998, o estado de São Paulo realizou um inquérito de âmbito estadual^13^. Seguiram-se iniciativas similares em alguns estados (Minas Gerais e Paraná, por exemplo) e em capitais, como Florianópolis, com o EpiFloripa^14^, e Goiânia^15^. Nesses inquéritos, planos amostrais específicos foram elaborados, ou foram feitas ampliações da amostra de alguma edição do SB Brasil.

### As metas oms-fdi em saúde bucal para o ano 2000

para avaliar a situação da cárie no Brasil, 10 anos após o “levantamento” de 1986, o MS realizou, em 1996, um inquérito – mantendo o “método combinado da OMS”, mas utilizando um plano amostral “simplificado” – sugerido pela OMS aos países com poucos recursos. Especialistas brasileiros consideraram essa simplificação inadequada ao país. A coleta de dados registrou dificuldades em vários municípios, inclusive capitais^2^. O MS buscava se preparar para realizar, no ano 2000, um inquérito epidemiológico para avaliar o atingimento das Metas em Saúde Bucal, fixadas para aquele ano pela OMS^16^.

As “Metas OMS-FDI/2000” haviam sido propostas à OMS em 1981 pela Federação Dentária Internacional (FDI)^17^. Embora a OMS tenha aceitado a proposição da FDI, logo surgiram críticas, argumentando-se que não fazia qualquer sentido a OMS propor metas globais^18^. Não obstante, as Metas OMS-FDI/2000 foram utilizadas no planejamento e na avaliação em saúde pública^19^, chegando-se até a cogitar novas metas para o ano 2010^20^. Contudo, jamais fixadas.

### A repibuco e a criação do projeto sb Brasil

em julho de 1996, 58 profissionais de odontologia, com interesse na epidemiologia da saúde bucal, reunidos em Curitiba durante o 12° Encontro Nacional de Administradores e Técnicos do Serviço Público Odontológico (Enatespo), constataram a insuficiente produção brasileira nessa área. Mesmo em relação à cárie dentária, não estavam disponíveis informações elementares sobre a situação da doença em importantes municípios, nas várias regiões do país. Para os demais problemas de saúde bucal, dados de base populacional inexistiam ou eram precários. Decidiram então propor ao MS a criação de uma Rede Brasileira de Epidemiologia em Saúde Bucal (Repibuco). Entre os objetivos estavam: criar e manter um banco de dados de abrangência nacional sobre epidemiologia da saúde bucal no Brasil; e contribuir para viabilizar a realização, no ano 2000, de um inquérito epidemiológico de base populacional e abrangência nacional, nessa área. Entretanto, o governo brasileiro, à época, descartou a proposta e optou por outra estratégia^21^.

Após a malsucedida experiência de 1996^2^, e já próximo do ano 2000, epidemiologistas vinculados à Repibuco, entidades e lideranças odontológicas indagaram o Ministério da Saúde sobre como o Brasil lidaria com as Metas OMS-FDI/2000. Esses questionamentos levaram o MS a instituir o projeto SB Brasil e fixar, para o ano 2000, sua 1^a^ edição, no âmbito do Sistema Único de Saúde (SUS).

A pesquisa SB Brasil 2000 teve uma implementação bastante difícil e foi concluída apenas em 2003^22^. Seguiram-se as edições de 2010 e de 2020.

### A edição 2023 do sb Brasil

Nenhum inquérito epidemiológico de base populacional é isento de dificuldades de várias ordens^23^, dificuldades estas que, em cada pesquisa, contribuem de algum modo para o aprimoramento de métodos e técnicas, fazendo avançar os conhecimentos nessa área. O SB Brasil, como projeto institucional vinculado ao SUS, constitui um bom exemplo. Da sua edição de 2003, resultou o aprimoramento dos planos amostrais^22^. Da edição de 2010, emergiram aspectos éticos derivados das características do treinamento de calibração dos examinadores, cuja técnica requer a repetição de exames na mesma pessoa^24^. Até essas edições, tanto o planejamento amostral quanto os aspectos éticos da calibração não vinham sendo suficientemente valorizados no SB Brasil. Na edição de 2023, esses aspectos receberam a devida atenção, e o resultado foi positivo.

A edição de 2020-2023 foi marcada por um contexto institucional federal desfavorável e pela pandemia de covid-19. Além disso, registrou importantes mudanças em relação às edições de 2003 e 2010, a respeito da gestão da pesquisa e alterações metodológicas com impacto no trabalho de campo. Tais mudanças resultaram em dificuldades de várias ordens, que merecem análise com vistas às próximas edições do SB Brasil. Embora neste artigo a ênfase recaia sobre a edição mais recente (2023) do projeto SB Brasil, deve-se levar em conta esse aspecto processual que caracteriza o SB Brasil e que constitui o objeto desta análise.

### Gestão da pesquisa

As edições concluídas em 2003 e em 2010 foram planejadas e executadas por uma coordenação técnico-científica constituída pelo MS, cujas atividades foram geridas pela Coordenação-Geral de Saúde Bucal (CGSB/MS) com apoio de entidades parceiras conveniadas com a pasta, exclusivamente para a gestão dos recursos financeiros alocados à pesquisa. Em 2000 o parceiro foi a Associação Brasileira de Odontologia (ABO) e em 2010, a Universidade de São Paulo (USP). Não obstante, não havia qualquer dúvida quanto ao SB Brasil ser uma pesquisa realizada pelo MS, no âmbito do SUS e, portanto, de responsabilidade compartilhada por todos os entes federativos^25^.

Todavia, a edição 2023 alterou esse padrão, com o MS outorgando à Universidade Federal de Minas Gerais (UFMG) não apenas a gestão financeira dos recursos da pesquisa, mas também a sua coordenação técnico-científica. Essa decisão teve implicações na imagem pública do SB Brasil, visto muitas vezes como “uma pesquisa da UFMG”. Não bastou que docentes vinculados à UFMG e a outras universidades, participantes do SB Brasil, tenham sempre negado se tratar de “uma terceirização da pesquisa”, pois foi desse modo que o SB Brasil 2023 foi percebido por muitos profissionais do SUS.

### Características metodológicas

Decisões envolvendo mudanças na padronização adotada pelo projeto SB Brasil, com base no “método combinado da OMS”, não são simples, pois implicam considerações metodológicas de várias ordens. Qualquer exclusão, como aconteceu com a fluorose dentária, ou inclusão, como ocorreu com o PUFA, deveria decorrer de análises exaustivas dessas implicações, incluindo na tomada de decisão vários especialistas com experiência epidemiológica. Como se deu o processo de decisão, com o desfecho que se conhece, é um assunto que merece mais reflexão.

No caso da exclusão da fluorose dentária, os principais argumentos que vieram a público são frágeis (baixa prevalência; concentração de casos em áreas endêmicas; não ser um problema relevante de saúde pública; a calibração ser muito difícil) e não justificam adequadamente a decisão, pois:

a) Inquéritos epidemiológicos são feitos para a contemporaneidade, mas se prestam, também, para comparações na linha do tempo. Assim, levar em conta apenas a prevalência atual de uma anomalia que tem despertado interesse em escala global não justifica a exclusão.
b) A concentração de casos em áreas endêmicas é uma característica da fluorose dentária “endêmica crônica”, mas esse não é o único tipo de fluorose dentária que põe em alerta sistemas de vigilância epidemiológica em vários países. Em áreas nas quais a exposição não se dá a uma fonte única de fluoretos, mas a múltiplas fontes, incluindo os cremes dentais, a fluorose dentária “iatrogênica endêmica”^26^ é a manifestação da anomalia que mais importa em inquéritos populacionais de abrangência nacional, como o SB Brasil.
c) Problemas de saúde pública são dinâmicos e, na linha do tempo, adquirem ou não relevância, dependendo de vários fatores que epidemiologistas não controlam. Por essa razão, mensurar (ao invés de não mensurar) deve ser a primeira opção nessas situações.
d) A calibração de examinadores para avaliar a presença de fluorose dentária (índice de Dean), embora reconhecidamente difícil, pode ser equacionada por treinamentos bem planejados e executados. Na edição de 2010 do SB Brasil, propusemos a calibração “in lux” para aprimorar o treinamento, buscando atenuar e superar a subjetividade inerente ao índice^24^. O resultado, porém, não foi bom. Entretanto, isso não se deveu, exclusivamente, à subjetividade do índice ou a dificuldades de calibração, mas, como registrou a literatura científica, a treinamentos de calibração mal planejados e executados e até a falsificação de dados no trabalho de campo, com necessidade de refazer a coleta de dados^25^.

A inclusão do índice PUFA representa o oposto do que ocorreu com a fluorose dentária: a inclusão não deveria ter ocorrido. Desde meados do século XX, quando a OMS reuniu epidemiologistas da saúde bucal para o esforço de padronizar métodos a serem empregados em inquéritos epidemiológicos, firmou-se um consenso sobre a relevância de estudos de base populacional levarem em conta a confiabilidade dos dados, derivados de sua precisão e validade. Nesse contexto, a sensibilidade, a especificidade e o valor preditivo requeridos dos instrumentos (índices) a serem utilizados são decisivos^27^. Por isso, ainda que haja grande interesse em estimar a prevalência de certas condições clínicas, deve haver cautela ao incluí-las em inquéritos de base populacional de grande abrangência, justificando adequadamente a inclusão^28^. Dados de prevalência relacionados com condições muitas vezes fugazes, como lesões de mucosas, por suas características, são considerados “problemáticos”^29^ e “difíceis de comparar com achados de estudos anteriores”^30^. Por suas restrições em estudos de prevalência, pois as discordâncias intra e interexaminadores são acentuadas, diminuindo a confiabilidade e tornando a calibração de examinadores mais complexa e muito exigente^28^, os instrumentos epidemiológicos desenvolvidos para avaliá-las deveriam ser empregados em estudos epidemiológicos longitudinais^30,31^. Além disso, a inclusão do PUFA correspondeu a uma duplicação desnecessária de dados, uma vez que o “método combinado” é suficiente para a avaliação epidemiológica da perda dentária e da “cárie não tratada”. Os autores que propuseram o PUFA^32^ repetem argumentos sobre “restrições ao índice CPOD” que, já em meados do século XX, foram rejeitados por epidemiologistas da saúde bucal com experiência de campo^33^.

### Aspectos do trabalho de campo

Além das dificuldades próprias dos territórios em que os dados são coletados, o trabalho de campo é fortemente afetado por dois componentes que requerem especial atenção: o treinamento de calibração e o conjunto das variáveis contidas no formulário, ou “ficha de exame”.

A emergência da pandemia de covid-19 impôs severas restrições ao trabalho de calibração, realizado com o emprego de Tecnologias Digitais de Informação e Comunicação (TIC)^34^. O emprego de TIC constituiu um avanço dessa edição do SB Brasil, aprimorando o treinamento e lidando com aspectos éticos dessa etapa de preparação para o trabalho de campo, notadamente o desconforto dos voluntários diante da necessidade de repetição dos exames^24^.

Entretanto, o principal problema que afetou o trabalho de campo foi o número de variáveis: 413. Esse número, excessivo, implica aumento de custos. Na base desse problema estão a inclusão de variáveis apropriadas a estudos longitudinais, mas não a estudos de prevalência (PUFA), e a duplicidade dos dados obtidos. Apenas dois exemplos de duplicidade: 1) o “traumatismo” dentário pode ser avaliado como apenas uma categoria (uma condição) da variável coroa dentária; e, 2) já a “oclusão dentária” pode ser avaliada com o emprego de apenas uma variável, com três categorias ou, se for o caso, utilizando-se apenas o Índice de Estética Dental (DAI - *Dental Aesthetic Index*). Embora a inclusão dessas variáveis na edição SB Brasil 2023 tenha sido baseada na 5^a^ edição do Manual da OMS, o resultado dessa decisão deveria ser priorizado no planejamento da edição 2030, cuja referência deveria ser a concepção sobre o que deve ser um inquérito como o SB Brasil, considerando a questão central desse tipo de pesquisa epidemiológica, referida desde meados do século XX^3^: “qual é a finalidade *deste* inquérito?”.

### Declaração de disponibilidade de dados

Todo o conjunto de dados que dá suporte aos resultados deste estudo, bem como o acesso aos relatórios das três edições do Projeto SB Brasil e demais relatórios institucionais mencionados, pode ser obtido junto ao Ministério da Saúde, Coordenação-Geral de Saúde Bucal.

### O que esperar do sb Brasil 2030

A viabilidade de uma edição 2030 do SB Brasil será avaliada nos próximos três anos, mas, ainda que nossa expertise aponte para um cenário positivo, a sua factibilidade, facilitada pelo acúmulo e pela consolidação da experiência brasileira nessa área, precisa ser analisada com brevidade. Um dos aspectos decisivos sobre a factibilidade é reduzir o número de variáveis compondo a “ficha de exame” com índices consolidados. Essa ponderação decorre da constatação de que, a cada edição, tem aumentado o número de variáveis e suas respectivas categorias, conforme mostra a Figura 2.

**Figura 2:** Número de variáveis dos bancos de dados produzidos por inquéritos populacionais epidemiológicos de saúde bucal no Brasil, em diferentes anos.

O Brasil se encontra, atualmente, em situação muito diferente da assinalada há 30 anos no Enatespo de Curitiba. Nesse período, o país produziu um acervo de inestimável valor sobre condições de saúde bucal da sua população. É compreensível, portanto, que os profissionais dessa área busquem qualificar ainda mais seus processos de investigação, desenvolver métodos, aprimorar técnicas. É mesmo tentador que se queira incluir nos protocolos de pesquisa o máximo de condições a observar no campo, mas é preciso parcimônia, cautela e prudência para não comprometer a factibilidade do SB Brasil e, assim, colocar em risco a sua continuidade como um valioso projeto institucional do SUS.

Nesse sentido, importa muito considerar custos, tempos, locais e técnicas de exame, características dos territórios e o que implicam para os planos amostrais, entre outros aspectos relacionados ao delineamento de cada edição do SB Brasil. Por essa razão, é crucial lidar adequadamente com a exigência da articulação clínica-epidemiologia. Não pode haver prejuízo da clínica, pois, como diz o adágio: “sem clínica, não há epidemiologia”. Contudo, a clínica não deve desconsiderar os requisitos não clínicos que se impõem à pesquisa epidemiológica de base populacional. Não são poucos, nem simples, esses requisitos.

Frequentemente, tem-se a expectativa de que inquéritos populacionais devem produzir uma imagem da realidade de saúde-doença de uma população, cuja nitidez e precisão sejam as melhores possíveis, equivalentes nos menores detalhes às de uma fotografia digital. Essa seria, então, a boa epidemiologia, a “expressão fiel da realidade”. Na maioria das vezes, porém, o máximo que se consegue é produzir um quadro impressionista. O alentador da pesquisa epidemiológica de base populacional é que, mesmo com as limitações de uma tela impressionista, a imagem que ela produz pode ser suficiente para fazer comparações, no tempo e no espaço, e decidir o que fazer. Por isso, inquéritos epidemiológicos são também instrumentos estratégicos para que gestores possam ajustar os modelos de atenção à saúde^12,23^. A Figura 3, com uma foto e uma pintura, ilustra essa perspectiva. Para um olhar qualificado, não faz falta uma fotografia digital, pois se consegue extrair da pintura impressionista, não obstante suas “deformações” da realidade, o que importa para avaliar situações e orientar a melhor tomada de decisões.

**Figura 3:** Os dois “fuscas vermelhos”: um capturado por uma câmera digital, e o outro pintado no estilo impressionista pelo artista plástico Sergio Romagnolo.

## CONSIDERAÇÕES FINAIS

A principal conclusão da presente análise é que a composição da coordenação técnico-científica de cada edição do projeto SB Brasil, e a gerência de suas atividades, incumbe ao Ministério da Saúde, bem como definir a finalidade desse tipo de inquérito epidemiológico, distinguindo-o de estudos epidemiológicos longitudinais em saúde bucal. Todos os profissionais envolvidos com a pesquisa, em qualquer âmbito, devem compartilhar a compreensão de que os dados a serem obtidos prestam-se a análises epidemiológicas e não a propósitos clínico-terapêuticos.

A experiência brasileira, ampla e consistente, precisa ser valorizada. O Brasil integra, nessa área, um grupo de países da vanguarda mundial, o que requer ações institucionais oportunas e pertinentes para não haver perda da expertise adquirida nas últimas décadas.

Aspectos relacionados com a viabilidade e a factibilidade dessa próxima edição foram analisados neste artigo, com a ênfase recaindo sobre a necessidade de reduzir o número de condições a observar, diminuindo o número de variáveis em relação à edição 2023 e cuidando para que apenas instrumentos epidemiológicos consolidados estejam incluídos. Inquéritos da magnitude do SB Brasil, cuja coleta de dados ocorre em territórios variados, sob distintas condições de execução, não são apropriados a experimentações, que podem impactar planos amostrais, custos, tempos, locais e técnicas de exame. Na análise feita neste artigo, enfatiza-se que decisões envolvendo mudanças na padronização construída no conjunto das edições do projeto SB Brasil, com base no “método combinado da OMS”, não são simples, pois têm consequências metodológicas de várias ordens, entre as quais as relacionadas com a sensibilidade, a especificidade e o valor preditivo, requeridos dos instrumentos epidemiológicos a serem utilizados, por suas implicações para a confiabilidade e validade dos dados.

## COMITÊ DE ÉTICA EM PESQUISA:

Tendo em vista as características deste artigo, do tipo ensaio, não houve necessidade de sua apreciação, específica, por Comitê de Ética em Pesquisa.
